# PB.35: Comparison of large-bore vacuum-assisted biopsy and surgical diagnostic excision biopsy in B3 breast lesions

**DOI:** 10.1186/bcr3535

**Published:** 2013-11-08

**Authors:** AH Tang, ND Forester

**Affiliations:** 1 Department of Breast Radiology, Royal Victoria Infirmary, Newcastle, UK

## Introduction

B3 lesion management is unclear. Despite varying malignancy risk, diagnostic excision was conventional, revealing malignancy in some and benign features in others. Large-bore vacuum-assisted biopsy (VAB, 7 to 8G) can enhance pathological certainty, providing reassurance of benignity, or identify co-existing malignancy, with many groups moving towards VAB to replace surgical excision biopsy. However, little research has addressed the accuracy of VAB compared with surgical biopsy in this situation.

## Methods

From November 2011 to May 2013, we incorporated VAB into the management of all B3 lesions identified by 10/14G biopsy. Excision biopsy was still performed on any lesion initially identified as B3 with atypia, unless upgraded by VAB. The VAB result and surgical excision outcomes were compared.

## Results

A total of 181 lesions were identified and considered for VAB. Atypia was present in 102 lesions. Sixty-five of 102 lesions had VAB, with 11 upgraded to malignancy. Of the 54/65 lesions not upgraded by VAB, 25 proceeded to excision biopsy, revealing DCIS in seven. A total 37/102 lesions did not have VAB (fibro-epithelial lesions/technical factors); malignancy was identified in five at excision biopsy. The overall malignancy rate was 23/102 (22.5%). Of VAB/excision biopsy discordant lesions, average tissue obtained at VAB was 2.4 g, compared with 2.9 g in concordant lesions (NS).

## Conclusion

VAB can increase preoperative detection of malignancy, but has an associated miss rate. Thirty per cent of the DCIS present in B3 lesions with atypia was missed by second-line VAB. This is important to appreciate as women are placed on surveillance programmes for B3 lesions following VAB, but without completion excision biopsy.

